# Cremation on the Battlefield

**Published:** 1895-06-15

**Authors:** 


### Cremation on the Battlefield.

The subject of cremation has a military as well as a civil side, for the enormous slaughter caused by modern projectiles so encumbers the ground with dead that the survivors from the perils of the battlefield are exposed to another almost equal danger from the festering corpses of those who have fallen. Many men interested in military hygiene have, then, turned their minds to cremation as a possible means of solving what is undoubtedly a great difficulty, and in the last two numbers of the *United Service Magazine* papers on the subject appear from the pen of A. G. Tait, of the Volunteer Medical Staff Corps. There can be no doubt of the very great evils which follow the hurried and partial burial which so frequently is given to the dead after a great battle; and if it should prove practicable to collect them into heaps and, by the aid of petroleum or any such fuel as might be locally available, to consume them to ashes, the sanitary benefits would be considerable. We may venture, however, to doubt whether in the manœuvres of an advancing or retreating army such a proceeding will be found feasible. Active warfare hinges too much upon rapidity of movement in the field, and this depends so entirely upon transport, that we can hardly expect precious means of transport to be used for carrying material for cremation. Every year the tendency will be more and more for moving armies to march on and leave the dead to bury their dead. But in sieges and in fixed encampments, where masses of men are congregated for considerable periods, the matter is very different, and cremation affords a solution of many difficulties in regard to the removal of refuse of all sorts. When thousands of men are camped out, depending for sanitation on improvised arrangements, their hygienic surroundings rapidly deteriorate, and their health suffers in proportion; and there can be little doubt that the health of a camp would be enormously improved by the systematic burning of all camp refuse, including contents of ashpits, waste from surgical operations, dressings, &c., and also including the bodies of the dead; but although we can also cordially endorse the advantages of cremation on the field of battle, we fear that to advocate such a proceeding for an advancing or retreating army is but a counsel of perfection.

